# Supplementary material for: Occurrence of Antithrombotic Related Adverse Events in Hospitalized Patients: Incidence and Clinical Context between 2008 and 2016
Source: J Clin Med. 2019 Jun 12;8(6):839. doi: 10.3390/jcm8060839 (PMC6617527; doi:10.3390/jcm8060839)
Supplement: Supplementary file 1 [file jcm-08-00839-s001.pdf]

Supplementary material

Table S1: Model parameters

**Parameter**	**Model: MRAE deceased OR (95% CI)**	**Model: MRAE discharged OR (95% CI)**	**Model: ARAE deceased OR (95% CI)**	**Model: ARAE discharged OR (95% CI)**
Year 2011/2012 (ref: 2008)	0.88 (0.63-1.22)	1.24 (0.73-2.09)	0.58 (0.30-1.13)	1.00 (0.38-2.64)
Year 2015/2016 (ref: 2008)	0.76 (0.54-1.08)	n/a	0.45 (0.23-0.88)	n/a
Academic Hospital (ref: tertiary teaching hospital)	0.99 (0.66-1.48)	1.54 (0.74-3.19)	1.37 (0.56-3.32)	0.24 (0.03-1.85)
General Hospital (ref: tertiary teaching hospital)	1.25 (0.91-1.72)	1.24 (0.69-2.23)	2.21 (1.12-4.37)	0.33 (0.11-0.96)
Gender (ref: female)	0.94 (0.73-1.21)	0.89 (0.55-1.43)	1.02 (0.61-1.71)	0.75 (0.28-1.99)
Age	0.99 (0.98-1.00)	1.01 (1.00-1.03)	1.01 (0.99-1.03)	1.07 (1.02-1.11)
Non elective admission (ref: elective admission)	0.74 (0.51-1.06)	1.06 (0.60-1.88)	0.64 (0.32-1.28)	0.84 (0.27-2.65)
Underwent surgery (ref: no surgery)	1.02 (0.70-1.49)	0.28 (0.11-0.70)	1.48 (0.74-2.98)	0.17 (0.03-0.99)
Admitted to surgical unit (ref: admitted to non-surgical unit)	0.75 (0.48-1.17)	0.45 (0.20-1.03)	0.97 (0.45-2.11)	1.32 (0.40-4.37)

n/a: parameter was not included in model

Table S2: Antithrombotic related adverse event example cases for each specific clinical situation

Case	Summary	Discharge status	Preventability score[a]
**Elevated INR**			
1	Intracranial haemorrhaging and decease following a fall incident together with elevated INR (>10).	Deceased	2-3
2	Intracranial bleed following a delayed response (±36 H) to elevated INR (9.3)	Deceased	2-3
3	Rectal bleeding following elevated INR. Prothrombin complex concentrate was prescribed but not signed off on medication chart and no INR verification performed.	Deceased	4-6
4	Haemoptysis with elevated INR (6.9) after starting Ceftriaxone.	Deceased	4-6
5	Gastrointestinal bleeding following elevated INR (16) and concomitant use of NSAID without PPI.	Deceased	4-6
6	Hematemesis following elevated INR (>8) which was only reversed with Prothrombin complex concentrate, therefor haematuria 2 days later after which Vitamin-K was administered.	Deceased	4-6
7	Bladder bleeding after radiotherapy for bladder cancer and elevated INR.	Deceased	4-6
8	Gastrointestinal bleeding following elevated INR (7.6) and no concomitant PPI for prednisone use.	Alive	4-6
9	Epistaxis following elevated INR due to concomitant use of Amoxicillin/clavulanic acid.	Alive	2-3
10	Variceal bleeding following elevated INR (>10) and decompensated liver cirrhosis.	Deceased	2-3
11	Subdural haematoma and decease following elevated INR (7.52) and delayed (72H) reversal.	Deceased	2-3
12	Pretibial haematoma following elevated INR (6.0) and Norfloxacin for urosepsis.	Alive	2-3
13	Epistaxis and gastrointestinal bleeding following elevated INR (5.4) and ASA and Clopidogrel use for ACS.	Deceased	4-6
14	TIA following reversal of elevated INR (6.4) with a too strong dose of Vitamin-K resulting in INR <1.	Deceased	4-6
15	Internal bleeding and shock following elevated INR (7.5) for which prescribed Vitamin-K was not administered.	Deceased	4-6
16	Gastrointestinal/variceal bleeding and decease following repeated elevated INR (7.5) and liver cirrhosis.	Deceased	4-6
**VTE prophylaxis**			
17	Pulmonary embolism following inadequate VTE prophylaxis.	Deceased	4-6
18	Pulmonary embolism following pneumonia and no VTE prophylaxis.	Deceased	4-6
19	Pulmonary embolism following metastasized pancreas carcinoma and no VTE prophylaxis.	Deceased	2-3
20	Deceased most likely due to pulmonary embolism following hip revision surgery with too low VTE prophylaxis dose.	Deceased	4-6
**Perioperative/periprocedural antithrombotic management**			
21	Severe postoperative bleeding and decease after hip replacement surgery while INR was elevated (10.4). Preoperative INR was not tested.	Deceased	4-6
22	Bladder bleeding following a correct ASA restart after interruption for nephroureterectomy.	Alive	1
23	Deceased due to a.basilaris thrombus following the interruption of ASA and Dipyridamole for hip surgery against Neurologists advise.	Deceased	2-3
24	Post procedural bleeding following polypectomy and correctly interrupted acenocoumarol.	Alive	1
25	Haemoptysis and haemorrhagic shock following oesophagoscopy with tumour biopsy for which Phenprocoumon was reversed with Prothrombin complex concentrate but INR was not verified pre-procedurally. (INR: 2.8 day after procedure).	Deceased	4-6

26	Ischaemic CVA following the preoperative interruption of Acenocoumarol without the use of LMWH bridging.	Deceased	2-3
27	Post procedural bleeding following liver biopsy and insufficiently reversed oral anticoagulants.	Deceased	4-6
**Disputed antithrombotic indication**			
28	Rectal blood loss from diverticulum while using ASA for questionable TIA indication	Alive	2-3
29	Gastric bleeding while using Clopidogrel for questionable ACS indication. No concomitant PPI prescribed.	Deceased	4-6
30	Recurrent kidney bleeding after kidney trauma and Dalteparin use.	Alive	4-6
31	Intestinal bleeding and decease following Nadroparin and possibly contraindicated Clopidogrel use.	Deceased	2-3
**Adverse drug reaction**			
32	Nausea and weight loss during Dipyridamole use after TIA. Symptoms disappeared after interruption of Dipyridamole	Alive	1
33	Heparin induced thrombocytopenia following UFH use. 48 hour delay before UFH was interrupted.	Deceased	4-6
**Patient related**			
34	Haematuria and sepsis following the manipulation of urine catheter by patient using ASA and Clopidogrel	Deceased	1
35	Found dead, possibly cardiac following the interruption of Clopidogrel and ASA, on patient initiative, 3 months after coronary stent placement.	Deceased	1
**Other**			
36	Haematoma and mucus bleeding while using ASA and clopidogrel after stent placement.	Alive	1
37	Gastro intestinal bleed during Dexamethasone for malignancy and Nadroparin use for pulmonary emboli.	Deceased	1
38	Persistent epistaxis while using ASA and Clopidogrel after stent placement	Alive	1
39	Retroperitoneal bleeding and decease following Acenocoumarol use for atrial fibrillation and CVA 1 week before.	Deceased	1
40	Subdural haematoma following a fall incident while using Acenocoumarol.	Deceased	4-6
41	Intracranial bleeding and decease while INR is in therapeutic range.	Deceased	1
42	Retroperitoneal bleeding and transfusion following continuous venovenous hemofiltration with UFH.	Deceased	1
43	Bleeding and severe swelling of hand around intravenous infusion needle following the use of various antithrombotic drugs.	Alive	1
44	Haemoptysis with bronchiecstasis following ASA initiation for TIA.	Alive	1
45	Bleeding from percutaneous coronary intervention entry site during antiplatelet use.	Deceased	1

INR International Normalized Ratio; NSAID Non-Steroidal Anti-inflammatory Drug; PPI Proton Pump Inhibitor; ASA Acetylsalicylic Acid; ACS Acute Coronary Syndrome; TIA Transient Ischaemic Attack; VTE Venous Thromboembolism; CVA Cerebrovascular Accident; LMWH Low Molecular Weight Heparin; UFH Unfractionated Heparin

[a] Preventability scores of 4 and higher were classified as potential preventable
